# District Nursing

**Published:** 1919-05-31

**Authors:** 


### THE LIFE OF A NURSE: ITS MORAL AND MATERIAL ADVANTAGES.

### IV. District Nursing.

District nursing was first started in Liverpool, about forty years ago, with the idea of providing the poor, in their own homes, with the skilled services of a trained nurse, who would give the necessary nursing attention to the sick person and at the same time bring the hygiene of hospital into the home. William Rathbone, the great Liverpool philanthropist, who was intensely interested in nursing and one of the first to encourage educated women to become probationers, gave the Home for the nurses and bore the whole of the initial expense. District nursing is now an important branch of the profession, established on a firm financial basis. The work has been perfected, and conditions have improved in every way. It is still hard work and perhaps, in normal times of peace, attended by more physical hardships and discomforts than any other life open to nurses when they have finished their training. This, however, is largely compensated for by the advantage of living so much in the open air. In the best associations, as in all good hospitals, the old ideals are still maintained. No woman should become a district nurse unless she has "the true spirit." It is work worthy of the best.

The work is full of interest. From a nursing point of view there is the never-failing charm and stimulus of helping to restore cases of acute illness, satisfaction in the growing skill in the management of chronic cases and in the ceaseless endeavours to relieve the sufferings of the incurable ones. There is the touching appeal of patients' pathetic gratitude. The human interest speaks for itself. The district nurse, coming not merely with advice or admonition, but obviously to render a service and supply a need, is generally a welcome visitor, admitted on terms of equality and with surprising rapidity to the innermost recesses of family life. If she has powers of observation and a sense of humour, there are splendid opportunities for obtaining a knowledge of human nature; her outlook on life will widen, her sympathies become broader and more tolerant. Though much of her work is sad, even at times depressing, the gay philosophy of the poor, who live from day to day, prevents it from ever being hopeless.

To become a district nurse it is necessary to be fully trained in general nursing. Some associations require also the C.M.B. certificate. Other experience is not necessary, but is useful if of the right kind. A ward sistership held for a few years after training gives greater knowledge of organisation, which is important in the arrangement of a big district. A short period of private nursing on a good staff, gives a knowledge of the ideal form of nursing as practised in a private house, which can be modified and adjusted in the restricted homes of the poor. After experience, however, should not be of too long duration, as it is essential that the candidate for district nursing should be fairly young, quite healthy, and not at all "set in a groove." The constant walking or cycling, the exposure to all weathers, are more easily overcome at the outset by the young than the middle-aged. The one essential qualification is the three years' certificate from a recognised training-school. District nurses, sometimes in towns, but more often in outlying country places, have to make decisions and act in cases of emergency where no doctor is available, and only the knowledge that a good training gives would justify them in taking the initiative on these occasions. District nursing requires also a short period of special training. Nurses who start the work knowing nothing of it, in isolated parishes, supported by a church or by private enterprise, however well-intentioned they may be, generally remain ineffectual and amateurish. The well-known and reliable associations give very careful training. There is a superintendent, herself fully trained and widely experienced, who inspects every district from time to time. Every new nurse is taken round her district for the first few weeks by the senior nurse or assistant superintendent. She is taught the methods used by the association, how to apply her own knowledge to the requirements of the district, how to carry out a system of antisepsis in unhygienic surroundings, how to bring to the sick in their own homes the comforts, the conveniences, and the cleanliness of hospital wards. She must learn to do all this in a tactful and helpful way without causing extra work and inconvenience for the rest of the family. She must learn to arrange her visits for the day with method and organisation, economising time, distance, and her own strength so that the work for each day is fairly equally proportioned. She must enter into the lives of her patients, be their friend and good counsellor, yet at the same time exercise the utmost reserve in all matters relating to herself and the association to which she belongs. She must learn to discriminate between the really poor and those who would take advantage of free services. She must have a knowledge of all the co-existing charities and relief-giving organisations; for although it is not advisable that the nurse herself should give her patients money or food, she should make every endeavour to obtain help for them if necessary.

Whilst making no impossible demands upon limited resources, being always ready to contrive and manage on the means at hand, the district nurse must set a certain standard, wage eternal war against dirt, waste, and ignorance, endeavouring to elevate the homes she visits. In this she can be public-spirited and co-operate in the work of health visitors, child-welfare workers, and school nurses. Most District Nursing Homes are now associated with the School Children's Treatment Centres, and supply a nurse for the minor ailments department, where affections of the eyes, ears, skin, etc., are treated. The nurse in this way gains a good deal of knowledge of the care and management of children and of the causes that undermine their health. The salaries of district nurses compare favourably with those of other branches of the profession, averaging now from £40 to £50 for those living in Homes, and from £100 to £120 for those working in country districts or small towns and having to provide for themselves. The higher posts to be obtained are superintendents, assistant superintendents, and county superintendents. The work is also a splendid foundation for any form of social work, such as that of a health visitor, a hospital almoner, or a probation officer, giving as it does an almost unique insight into the lives of the poorer classes.

